# Comments on “Negative effect of varicocele on sperm mitochondrial dysfunction: A cross-sectional study”

**DOI:** 10.18502/ijrm.v22i7.16976

**Published:** 2024-09-12

**Authors:** Elaheh Sanjari, Hadi Raeisi Shahraki

**Affiliations:** ^1^Student Research Committee, Shahrekord University of Medical Sciences, Shahrekord, Iran.; ^2^Department of Epidemiology and Biostatistics, Faculty of Health, Shahrekord University of Medical Sciences, Shahrekord, Iran.

##  Dear Editor

Recently, Elahi et al., published an article entitled above and concluded that the varicocele negatively affects sperm concentration, motility, morphology, sperm DNA fragmentation index, mitochondrial membrane potential level, and adenosine triphosphate (1). However, we believe that the conclusion is doubtful due to the following reasons:

1. Talking about any causality effect in the cross-sectional studies is forbidden, because exposure and outcome are assessed at the same time and may be susceptible to reverse causality (2).

2. There is no confounder adjustment in the results section. For example, the paternal age is one of the most important confounders. Findings of a meta-analysis showed a 3–22% decrease in semen volume and 3–37% in sperm motility among men over 50 yr (3), but we did not find any age adjustment.

3. Statistically significant does not reflect any information about the effect of varicocele.

4. It lacks any statistical analysis to show the prediction power, discrimination power, or variable importance. The performed statistical analysis (Mann-Whitney U test and Spearman correlation) does not infer the effect of varicocele on sperm mitochondrial dysfunction.

Apart from the points discussed in this note, which we hope will be useful to the researchers, we would like to thank Elahi et al., for sharing their valuable article and in-depth investigation and analysis with us. In conclusion, the interpretations of findings must be regarded with caution.

##  Conflict of Interest

Nothing to declare.

## References


ElahiM
HojatiV
HashemitabarM
AfroughM
KargarHM
DastoorpoorM
Negative effect of varicocele on sperm mitochondrial dysfunction: A cross-sectional studyInt J Reprod BioMed2023213233323726054910.18502/ijrm.v21i4.13271PMC10227351
SavitzDA
WelleniusGA
Can cross-sectional studies contribute to causal inference? It dependsAm J Epidemiol20231925145163523193310.1093/aje/kwac037
KiddSA
EskenaziB
WyrobekAJ
Effects of male age on semen quality and fertility: A review of the literatureFertil Steril2001752372481117282110.1016/s0015-0282(00)01679-4

##  Authors' Reply

Regarding the dispute in the letter about an article entitled “Negative effect of varicocele on sperm mitochondrial dysfunction: A cross-sectional study” (1). The critics believe that the conclusion is doubtful due to the reasons mentioned in the abovementioned “Letter to the Editor”; the authors answers are as follows.

In response to the first question, it is necessary to mention that obviously, a cross-sectional study is not always sufficient to determine any causality effect. However, it often paves the way for other investigations to measure the effects of causal parameters. This means it can prepare an appropriate scenario to address relationships between measures obtained from cross-sectional studies as well as it may be building an outline from prevalence ratio to cumulative incidence ratio (2). A cross-sectional study can be somewhat similar to a case-control study if we exclude as many confounding variables as possible. Although, the dimension of time does not make it suitable for measuring short-effect diseases, with a shorter duration of symptoms. In contrast to varicocele, case selection was based on including patients who did not undergo surgery, and therefore, they are usually associated with long-term outcome consequences. So, in the authors' opinion, it is a suitable design to measure the probability effects of causal parameters or assess the prevalence of outcomes/exposures in clinic-based samples. It is important to demonstrate through a multivariable cross-sectional study to clarify the effect of risk factors (i.e., mitochondrial membrane potential) for disease (i.e., varicocele). On the other hand, it can usually be conducted as a relatively faster and more reliably inexpensive study for Ph.D. thesis (3).

In response to the second question, it is stated that “there is no confounder adjustment in the results section”. It should be noted that semen quality certainly declines with age in men, but we adjusted for age, weight, and many other confounding factors in this study. For example, the mean age in the varicocele group was 31.78 
±
 7.54 yr and in control group were 31.10 
±
 15.12 yr, this means that no significant age difference was observed between the 2 groups. The following sentences were added in the first paragraph of the results section.

“The mean age for both the varicocele and control groups was similar (31.78 
±
 7.54 vs 31.10 
±
 15.12, respectively), with no significant difference between them (p = 0.777). Likewise, no significant difference was observed in BMI between the varicocele and control groups (24.97 
±
 2.85 vs 23.95 
±
 3.01, respectively; p = 0.085)”.

In response to the next question, it is necessary to mention that significantly does not necessarily reflect the significant impact of varicocele effects in the population; but mainly it reflects the true outcome of a research design with limited sample size, degree of validity, reliability, and sensitivity of implemented tests in a clinical research study that make it a suitable for Ph.D. thesis. Also, critics have stated that there is a lack of statistical analysis to show the prediction power, discrimination power, or variable importance. Initially, this study did not aim to investigate the impact of varicose veins. Instead, its primary objective was to explore the correlation between parameters within 2 distinct subgroups. Therefore, there was no need to evaluate the study's power to ascertain the effect. Additionally, the tests conducted in this study were univariate and in instances where the data distribution deviated from normality,

non-parametric tests were employed, despite their relatively lower power compared to parametric tests. Since the study's main focus was examining correlations, there was no need to determine the tests' power. Table II was modified according to the above description and was added to the updated version of article (https://doi.org/10.18502/ijrm.v21i4.13271).

##  Editorial

Thanks to Dr. Hadi Raeisi Shahraki and Colleagues for their comments on the article that was published in our journal issued 2023; 21: 323-332. The modified version of this article will be uploaded in our electronic version (July 2024).

